# Rabies in Foxes, Aegean Region, Turkey

**DOI:** 10.3201/eid1510.090203

**Published:** 2009-10

**Authors:** Ad Vos, Conrad Freuling, Seza Eskiizmirliler, Hikmet Ün, Orhan Aylan, Nicholas Johnson, Semra Gürbüz, Winfried Müller, Necdet Akkoca, Thomas Müller, Anthony R. Fooks, Haluk Askaroglu

**Affiliations:** IDT Biologika GmbH, Dessau-Rosslau, Germany (A. Vos); Friedrich-Loeffler-Institute, Wusterhausen, Germany (C. Freuling, T. Müller); Bornova Veterinary Control and Research Institute, Izmir, Turkey (S. Eskiizmirliler, N. Akkoca); Etlik Central Veterinary Control and Research Institute, Ankara, Turkey (H. Ün, O. Aylan); Veterinary Laboratories Agency, Weybridge, UK (N. Johnson, A.R. Fooks); Technical Assistance for Control of Rabies Disease, Ankara (S. Gürbüz, W. Müller); Ministry of Agriculture and Rural Affairs, Ankara (H. Askaroglu)

**Keywords:** Rabies, dog, fox, spillover, Turkey, viruses, dispatch

## Abstract

At the end of the 1990s in the Aegean region of Turkey, rabies rapidly spread among foxes. This spread likely resulted from spillover infection from dogs and led to increased rabies cases among cattle. To control this outbreak, oral rabies vaccination of foxes has been used.

In Turkey, dog-mediated (spread by dogs as host species) rabies dominates the epidemiology of rabies (*1*). During 1990–2000, a total of 2,856 rabies cases were reported from Turkey; dogs (*Canis lupus familiaris*) accounted for 78% of reported cases, whereas wildlife accounted for only 1.6% (data from 44 issues of Rabies Bulletin Europe, available from www.who-rabies-bulletin.org). In the past decade (1998–2007), however, an increasing number of rabies cases in foxes (*Vulpes vulpes*) have been reported from the Aegean region in western Turkey. Rabies in foxes has been reported incidentally from other regions in Turkey, especially from the central and eastern parts. Rabies cases in foxes have been considered to be rare, dead-end, spillover events from rabid dogs and to have no epidemiologic significance. However, surveillance data from most of these regions are limited; therefore, whether rabies in wildlife occurs independently from rabies in dogs is unknown. Sufficient data are available for the Aegean region, and phylogenetic studies have concluded that rabies recently spilled over from domestic dogs to foxes in this area (*2*).

The Aegean region is characterized by mountain ranges, except for the coastal plains, where most of the human population is concentrated and where ≈3.5 million persons live in Turkey’s third largest city, Izmir. This economic and industrial center lies in a predominantly agricultural area. Before 1999, rabies in the Aegean region was predominantly mediated by dogs, and no clear movement from an urban focus was noted. Most cases were observed in and around the city of Izmir. The number of rabies cases had decreased notably in the Aegean region, from 137 cases in 1988 to only 2 cases in 1995, after which the number started to increase again (*1*).

To determine more about the epidemiology of this disease, we analyzed the spatial and temporal incidence of rabies in 8 provinces of the Aegean region (Figure 1) during 1998–2007. We emphasized the shift from dog-mediated to fox-mediated rabies and the consequences to the disease profile in this area.

**Figure 1:**
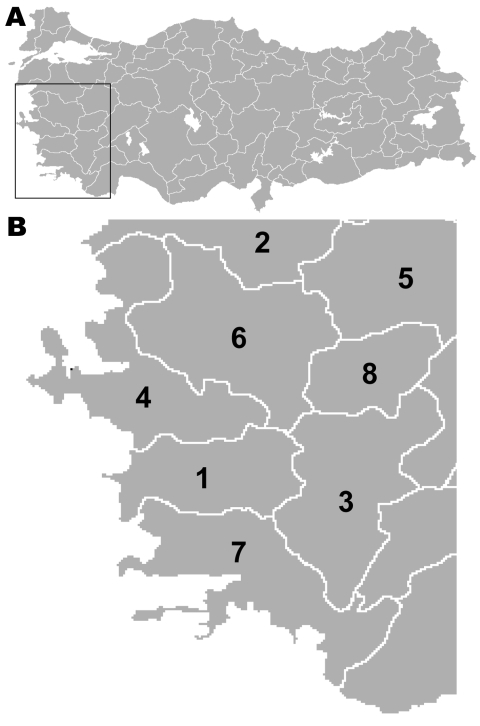
A) Map of Turkey showing location of Aegean region (box). B) The 8 provinces in the Aegean region of Turkey that were studied for spatial and temporal incidence of rabies during 1998–2007. 1, Aydin; 2, Balikesir; 3, Denizli; 4, Izmir; 5, Kütahya; 6, Manisa; 7, Mugla; 8, Usak.

## The Study

During the study period, animals from the Aegean region suspected of having rabies were submitted for rabies diagnosis to the Bornova Veterinary Control and Research Institute in Izmir. Routine rabies diagnosis was based on detection of Negri bodies, followed by fluorescent antibody test results; for negative samples, the mouse inoculation test was also performed (*3*). From 1998 through 2007, a total of 3,737 specimens were submitted; of these, 118 samples could not be examined because the samples were of poor quality (Table 1). Analysis of the data for the 3 major animal species submitted (cattle, dogs, and foxes) during this period showed dramatic changes in the number of rabies cases for each of these species (Table 2). In 1998, no rabies in foxes was reported, and dogs clearly dominated rabies submissions. However, in subsequent years, rabies emerged in foxes; this emergence coincided with an increased number of rabid cattle reported and a decreased number of rabid dogs. The annual number of rabid dogs correlated only weakly with total number of dogs submitted for testing; coefficient of determination (R^2^) was 0.56. However, this correlation was markedly higher for cattle and foxes (R^2^ = 0.99 for both species). This finding indicates that the lower number of rabid dogs did not result only from decreased surveillance for this species.

**Table 1:** Rabies testing results for specimens submitted to Bornova Veterinary Control and Research Institute, Izmir, Turkey, 1998–2007

Species	No. positive/total no. tested (% positive)
Horses	5/17 (29)
Donkeys	4/14 (29)
Goats	29/46 (63)
Sheep	27/49 (55)
Cattle	605/758 (80)
Cats	46/782 (6)
Dogs	327/1,581 (21)
Hamsters	0/49 (0)
Rabbits	1/18 (6)
Mice and rats	0/67 (0)
Squirrels	0/11 (0)
Foxes	165/174 (95)
Jackals	2/2 (100)
Wolves	0/2 (0)
Badgers	1/2 (50)
Mustelids	13/18 (72)
Wild cats	0/1 (0)
Bats	0/5 (0)
Humans	6/8 (75)
Other	0/15 (0)

Total

1,231/3,619 (34)

**Table 2:** Rabies testing results for 3 most commonly affected species, Aegean region, Turkey, 1998–2007*

Year	Species									Total†	
	Dogs			Cattle			Foxes				
	Pos	Neg		Pos	Neg		Pos	Neg		Pos	Neg
1998	67	102		3	4		0	1		76	177
1999	78	104		13	5		3	1		106	193
2000	39	158		7	5		10	1		72	281
2001	30	129		66	16		22	0		139	252
2002	30	125		236	24		44	0		339	247
2003	11	142		100	13		24	0		154	251
2004	12	124		49	24		9	1		74	248
2005	17	126		55	18		14	1		95	234
2006	17	114		39	10		20	1		89	229
2007	26	130		37	34		19	3		87	277

*Pos, positive; Neg, negative. †Includes all species tested.

**Figure 2:**
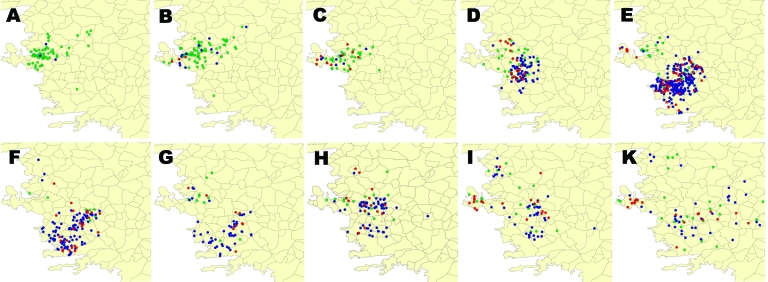
Location of rabies cases in the 3 most affected species in Aegean region by year, 1998–2007. Red, red foxes; green, dogs; blue, cattle.

From 1998 through 2000, almost all cases in the 3 most affected species were reported from the area between the cities of Izmir and Manisa (Figure 2), and the epizootic progressed in a wave-like fashion southward; in 3 years, the area of rabies cases moved ≈150 km. In 2003, the southeastward movement of the rabies epidemic slowed and, in 2004, appeared to halt. In 2005, cases again increased in the northern part of Aydin Province, which borders Izmir Province. In 2006, no clear movement of this outbreak was observed.

The northern Aegean region may not be a suitable habitat for foxes and therefore may not have supported a sufficient number of foxes for an epizootic. Reliable data on fox density or even the number of foxes shot are lacking. Several cases of rabies in foxes were reported in 2006 from the area where the fox outbreak had started 7 years earlier. In 2007, rabies in foxes was still reported from this area, and the outbreak in Aydin had moved eastward and established itself in the neighboring province of Denizli.

## Conclusions

The high number of free-roaming dogs and the low vaccination coverage of the dog population would have provided ample opportunities for infected dogs to transmit the virus to foxes. Hence, foxes (or a fox) in the Izmir area are assumed to have become infected, and rabies then spread in the fox population. The close association of the sequences of rabies virus isolates from dogs and foxes in this area supports a recent transfer (*2*). However, the phylogenetic data provide no indication of the direction of virus transmission. The lack of reported rabies in a fox in this area since 1986 suggests that dogs transferred the virus to foxes, leading to the present rabies outbreak, not the reverse.

Since 1999, rabies has moved predominantly south and southeast at ≈40–50 km per year from the area where the first cases in foxes were reported. This movement was associated with increases in the number of rabies cases in foxes and cattle until 2003, when the number of cases sharply declined after mass vaccination of cattle. Since 2003, the numbers of rabid dogs and rabid foxes reported each year has remained approximately the same. However, the number of dogs submitted for rabies diagnosis is ≈9× higher than the number of foxes submitted. Furthermore, only 20% of the dogs tested were rabies virus–positive, compared with almost all (95%) of the foxes; therefore, the true number of rabid foxes can be assumed to exceed the number of rabid dogs.

Although during the 10 years of the study period, 6 cases in humans in the study area were reported, none were linked to foxes. Hence, the public health risks associated with rabid foxes are relatively small compared with those associated with rabid dogs. However, if not eliminated, rabies in foxes will form a reservoir for disease persistence. A high number of rabies cases in cattle causes an economic loss. Mass vaccination of cattle reduces these losses but does not solve the problem. After the initial epizootic, the disease seems to have become endemic to the Aegean region. Preventing the spread of rabies to foxes in unaffected areas is crucial. Therefore, to control the present outbreak, a campaign to orally vaccinate foxes against rabies in the affected provinces of Turkey was initiated during February 2008 and conducted again in 2009.

## References

[1] Akkoca N, Economides P, Maksoud G, Mestom M. Rabies in Turkey, Cyprus, Syria and Lebanon. In: King AA, Fooks AR, Aubert M, Wandeler AI, editors. Historical perspective of rabies in Europe and the Mediterranean basin. Paris: Office International des Épizooties; 2004. p. 157–169.

[2] Johnson N, Un H, Vos A, Aylan O, Fooks AR. Wildlife rabies in western Turkey: the spread of rabies through the western provinces of Turkey. Epidemiol Infect. 2006;134:369–75. 10.1017/S0950268805005017 16490142 PMC2870393

[3] Meslin FX, Kaplan MM, Koprowski H. Laboratory techniques in rabies, fourth ed. Geneva: World Health Organization; 1996.

